# The CAPER studies: five case-control studies aimed at identifying and quantifying the risk of cancer in symptomatic primary care patients

**DOI:** 10.1038/sj.bjc.6605396

**Published:** 2009-12-03

**Authors:** W Hamilton

**Affiliations:** 1 Department of Community Based Medicine, NIHR School for Primary Care Research, University of Bristol, 25-27 Belgrave Road, Bristol BS8 4AA, UK

**Keywords:** diagnosis, primary health care, predictive values

## Abstract

**Background:**

This paper reviews the background to five primary care case-control studies, collectively known as the CAPER studies (Cancer Prediction in Exeter). These studies, on colorectal, lung, prostate and brain tumours, sought to identify the particular features of cancer as reported to primary care. They also sought to quantify the risk of cancer for symptoms and primary care investigations, both individually and paired together.

**Methods:**

Two studies were on colorectal cancer: the former with 349 cases used hand searching and coding of entries, while the latter obtained 6442 cases from a national electronic database. The lung and prostate studies had 247 and 217 cases, respectively, and used manual methods. The brain study also used a national electronic database, which provided 3505 cases.

**Results:**

Generally, the symptoms matched previous series from secondary care, though the risks of cancer, expressed as positive predictive values, were lower. Rectal bleeding in colorectal cancer, and haemoptysis in lung cancer both had positive predictive values of 2.4%. The risk of a brain tumour with headache was one in a thousand.

**Interpretation:**

The results identify areas where current guidance on urgent referral for investigation of suspected cancer could be improved.

Several papers in this supplement refer to the relatively poor cancer outcomes in the United Kingdom (Verdecchia *et al*, 2007). Part of the underperformance relates to delays in diagnosis, which may account for over 5000 extra deaths annually (Richards, 2009). Diagnostic delays may be reduced in three main ways: by screening, by earlier presentation when symptoms are experienced (the so-called ‘patient delay’), or by better identification of cancer in the symptomatic patient. Although screening reduces mortality in several cancers, current UK programmes will identify only one-tenth of the total number of UK cancers, leaving the other 90% to present with symptoms (Goodyear *et al*, 2008; Hamilton, 2009). This is usually to primary care (Hamilton and Peters, 2007).

Despite the importance of cancer as a cause of death, at the level of the individual general practitioner (GP) it is rare (Summerton, 2002). Every year, a full-time GP will have one patient diagnosed with each of the four common cancers (breast, lung, colon and prostate). All other cancers (except skin) are considerably less common – for instance, a new ovarian cancer is diagnosed every 5 years per GP. Therefore, GPs obtain little personal experience of cancer diagnosis, although few days will pass without a patient consulting with a symptom that could represent malignancy. This is the crux of the problem: how does a GP identify the one patient with cancer from among the many who do not? The problem can be split into two interrelated questions. The first is simple: could this symptom represent cancer? Although almost any symptom could portend an underlying cancer, in practice GPs use a relatively short checklist of ‘red-flag’ symptoms. When a patient describes one of these selected symptoms, the second question is how likely is cancer? Symptoms may pass the first test, but often fail the second. For example, GPs know that constipation is a symptom of colorectal cancer, yet rarely actually investigate for it, as the risk of cancer is deemed to be too small to warrant testing. This is the rationale for national guidance, particularly that from the National Institute for Health and Clinical Excellence (NICE, 2005). However, the evidence base for such guidance was poor, at least in terms of primary care studies (Hamilton and Sharp, 2004).

To address these two practical primary care questions, a series of studies was designed, with two main objectives: to identify symptoms of common cancers that are presented to primary care, and to quantify these symptoms. These studies mostly took place in Exeter, Devon, UK, and thus the acronym CAPER was chosen, standing for Cancer Prediction in Exeter. Five studies have been completed, and are published. They relate to colorectal (Hamilton *et al*, 2005b), lung (Hamilton *et al*, 2005a), prostate (Hamilton *et al*, 2006) and brain tumours (Hamilton and Kernick, 2007), with the fifth repeating colorectal, but focussing on anaemia (Hamilton *et al*, 2008). A sixth, on ovarian cancer has recently been published (Hamilton *et al*, 2009b). A seventh study, on the features of metastatic cancer, has begun.

## Materials and methods

### Study design

The gold standard method of identifying and quantifying the risk of cancer (or any outcome) associated with a symptom is a prospective study. A conventional prospective study would be impossibly large to conduct in primary care, given the relative frequency of symptoms, coupled to the relative rarity of new cancer diagnoses. Furthermore, the list of symptoms for study would have to be determined in advance, meaning any previously unrecognised diagnostic feature of cancer would be missed. For the CAPER studies, case-control methods were chosen. The main concern with such studies is the potential bias from patient recall. Patients interviewed after diagnosis may remember features they consider to be important, whereas the comparison group, who may have had similar symptoms, may not retain such memories. This bias can be eliminated by using data collected before the diagnosis was made – in this case using GP records.

### Case identification and control generation

For the first three studies (henceforth called the Exeter studies), cases were identified from the local cancer registry, supplemented by computerised searches at each participating surgery. Once the inclusion and exclusion criteria were applied (these largely related to doubtful diagnoses, or to missing records) five age-, sex- and practice-matched controls were generated from the doctors' computers. Controls who had not consulted in the 2 years before the diagnosis date of their matched case were rejected. Similar methods were used in the two national electronic database studies (henceforth called the electronic studies), other than the cases being identified by searches on the GP records, and seven controls being available (this being the standard number offered by the databases).

The entire medical record for 2 years was coded by trained research assistants using the International Classification of Primary Care-2 – the most symptom-based of the coding systems (Okkes *et al*, 2002). In the electronic studies, only 1 year of records was examined, and pre-selected symptoms were identified.

### Analytical methods

The studies had similar analytical methods. All variables associated with cancer in univariable conditional logistic regressions (usually around 100 variables) entered multivariable analyses. The multivariable analyses generally reduced the number of variables to around 10, all of which were independently associated with cancer, mostly at a *P*-value <0.001. For these features, a univariable likelihood ratio was calculated.

Note: *Likelihood ratio*: This is simpler than it sounds. This is the chance of a patient with cancer having a symptom of the cancer, divided by the chance of a patient without cancer having the same symptom. For example, in lung cancer, 20% of cases had reported haemoptysis, whereas 1.5% of controls had done so. Thus, the positive likelihood ratio was 20/1.5=13.

*Positive predictive value*: This is the chance of a patient having the disease of interest when they have reported the symptom. For the same example, a person reporting haemoptysis to their GP has a 2.4% chance of the symptom being due to an underlying lung cancer.

There is a relationship between these two metrics. The positive predictive value (PPV) for the symptom is the annual incidence of the relevant cancer multiplied by the likelihood ratio for the symptom. Strictly, it is slightly more complicated than that, in that the PPV and the incidence have to be expressed as odds for the calculations, and then converted back to percentages, but the essential point is that the larger the likelihood ratio, the higher the positive predictive value.

From this likelihood ratio, and using the incidence of cancer and Bayes' theorem, a PPV for each variable could be calculated (Knottnerus, 2002). Calculation of positive predictive values was then repeated using pairs of symptoms, and in different strata, where the size of the databases allowed it.

## Results

Details of the data sources for the studies are summarised in Table 1. Identification of cases in the Exeter studies was generally straightforward, with a high percentage of cases supported by histological proof. This was not available for the two electronic studies. The incidence of cancer in the Exeter studies was very similar to national figures, whereas it was slightly below national figures for the electronic studies, suggesting that some cancer diagnoses were missing from the electronic databases. In all studies, the age ranges were similar to national figures.

Consultation rates and symptom reporting were significantly higher for cases before diagnosis, particularly in the 6 months immediately before diagnosis. The symptoms independently associated with cancer after the multivariable analyses are summarised in Table 2. These are ranked by likelihood ratio, showing that the symptom with the strongest association with cancer is not necessarily the most common symptom in cases. Lung cancer symptoms had markedly lower likelihood ratios when compared with the other cancers – other than that for haemoptysis. This reflected the frequency of lung cancer symptoms in the non-cancer population. Thus, 65% of lung cancer cases had reported cough (with 43% reporting it a second, and 28% a third time before diagnosis), yet the likelihood ratios remained very low. Even lower was the risk of a brain tumour in a patient presenting to primary care with a new-onset headache, at one in a thousand.

Abnormal primary care investigations are summarised in Table 3. No primary care tests were found to be associated with brain tumours. The risk of colorectal cancer with anaemia was very high for the values recommended by NICE as warranting referral: a haemoglobin value of <10 g dl^−1^ in women equating to an 8% risk, and of <11 g dl^−1^ in men, a 13% risk (Hamilton *et al*, 2008).

Three unexpected associations were noted: between a raised glucose level and colorectal cancer, between thrombocytosis and lung cancer, and between impotence and prostate cancer. Diabetes is a well-accepted risk factor for colorectal cancer (Larsson *et al*, 2005). Even so, it was a surprise that a raised blood sugar remained significant in the final multivariable models including symptoms. Thrombocytosis as a feature of lung cancer had been described once before in a hospital series of patients awaiting investigation for possible cancer, but not previously in primary care (Pedersen and Milman, 2003). Finally, impotence was found to have a strong association with a future diagnosis of prostate cancer, with a PPV of 3%, a previously unreported finding.

**Figure 1:**
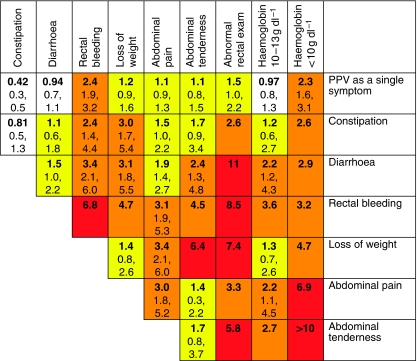
Positive predictive values (%) for colorectal cancer for individual features, repeat presentations and for pairs of features (in the context of a background risk of 0.25%). *Notes*: (1) The top row (bold) gives the PPV for an individual feature. The cells along the diagonal relate to the PPV when the same feature has been reported twice. Thus, the constipation/constipation intersect is the PPV for colorectal cancer when a patient has attended twice (or more often) with constipation. Other cells show the PPV when a patient has two different features. (2) The top figure in each cell is the PPV. It has only been calculated when a minimum of 10 cases had the feature or combination of features. The two other figures are the 95% confidence intervals (CIs) for the PPV. These have not been calculated when any cell in the 2 × 2 table was below 10. For haemoglobin <10 g dl^−1^ with abdominal tenderness, no controls had this pair. It was scored as a PPV of >10%. (3) The yellow shading is when the PPV is above 1%. The amber shading is when the PPV is above 2%, which approximates to a risk of colorectal cancer of eight times normal. The red shading is for PPVs above 5.0% approximating to a risk of 20 times normal.

**Figure 2:**
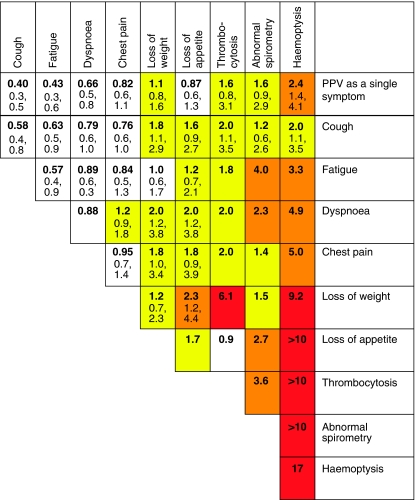
Positive predictive values (%) for lung cancer for individual risk markers, and for pairs of risk markers in combination (against a background risk of 0.18%). *Notes*: (1) The top row (bold) gives the PPV for an individual feature. The cells along the diagonal relate to the PPV when the same feature has been reported twice. Other cells show the PPV when a patient has two different features. (2) The top figure in each cell is the PPV. It has only been calculated when a minimum of 10 cases had the feature or combination of features. The two other figures are the 95% CIs for the PPV. These have not been calculated when any cell in the 2 × 2 table was below 10. (3) The yellow shading is when the PPV is above 1%. The amber shading is when the PPV is above 2%. The red shading is for PPVs above 5.0%.

**Figure 3:**
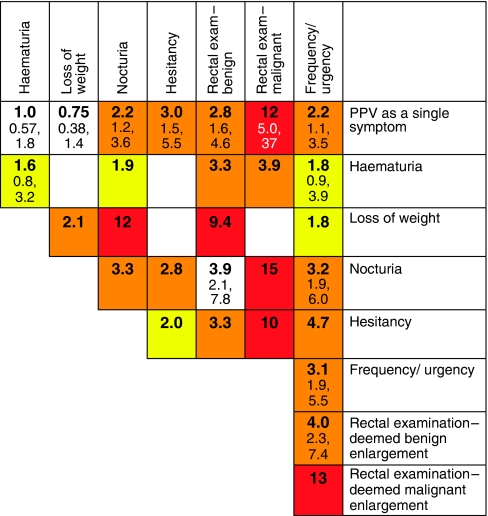
Positive predictive values (%) for prostate cancer for individual features, repeat presentations and for pairs of features (against a background risk of 0.35%). *Notes*: (1) The top row (bold) gives the PPV for an individual feature. The cells along the diagonal relate to the PPV when the same feature has been reported twice. (2) The top figure in each cell is the PPV. It has only been calculated when a minimum of 10 cases had the feature or combination of features. The two other figures are the 95% CIs for the PPV. These have not been calculated when any cell in the 2 × 2 table was below 10. (3) The yellow shading is when the PPV is above 1%. The amber shading is when the PPV is above 2%. The red shading is for PPVs above 5.0%.

**Figure 4:**
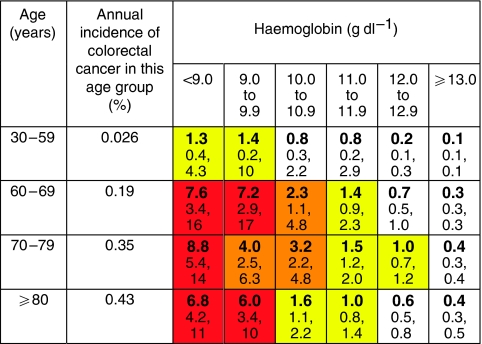
PPV (%) for colorectal cancer of haemoglobin result taken in primary care, in males by age (with 95% CIs). *Note*: Incidence data, Cancer Research UK, 2003.

**Figure 5:**
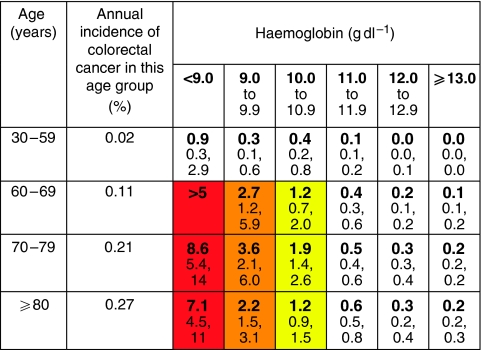
PPV (%) for colorectal cancer of haemoglobin result taken in primary care, in females by age (with 95% CIs). *Note*: Incidence data, Cancer Research UK, 2003.

Positive predictive values were calculated for individual symptoms, pairs of symptoms and symptoms reported for a second time in primary care. These values are shown in Figure 1 (colorectal), Figure 2 (lung), Figure 3 (prostate), and Figures 4 and 5 (anaemia).

## Discussion

The five studies largely confirmed that the symptoms of cancer listed in medical textbooks were relevant in primary care, and were often reported months before diagnosis. The new information was the relative importance of the symptoms, and the risk of cancer posed by each one. Current UK referral guidance suggests particular symptoms for urgent referral, without any explicit rationale given for the choice. Now there are risk estimates for the symptoms of some common cancers, the choice of symptoms can be placed on a stronger footing.

Some important points emerged for individual cancers. For colorectal cancer, it was clear that a policy of concentrating on rectal bleeding will only yield a small return, in that only 42% of cases experience this symptom, and referrals are made quickly (Barrett *et al*, 2006). Furthermore, the threshold haemoglobin values recommended for referral of iron deficiency anaemia equated to very high risks – at least when compared with the risks inherent in the other symptoms deemed worthy of referral. Perhaps the most important concept to arise from the colorectal studies was the ‘low-risk-but-not-no-risk’ symptom. Most patients with colorectal cancer never have a high-risk symptom such as rectal bleeding or severe anaemia. Thus, most do not qualify for urgent referral, explaining in part why so few colorectal cancers are diagnosed through the 2-week wait clinics (Thorne *et al*, 2006; Rai and Kelly, 2007). Not surprisingly, mortality is highest in patients whose first symptom is abdominal pain, and lowest in those with rectal bleeding (Stapley *et al*, 2006a). Thus, a scoring system – the CAPER score – has been derived to try and identify those most at risk of cancer, when they only have a low-risk symptom (Hamilton, 2007; Khan and NCRI Colorectal Clinical Studies Group, 2009). It encourages GPs to identify other symptoms when a patient attends with a low-risk symptom such as diarrhoea, and a score is derived from the symptom list, plus the haemoglobin level. Initial modelling in a second data set seems very promising, suggesting that there may be a way of identifying cancer in those who are currently not prioritised for investigation.

In lung cancer, a concern was that haemoptysis – this time the only symptom with a risk above 2% – was also relatively rare. Thus, to expedite diagnosis of lung cancer, the focus will have to be on the softer symptoms, such as dyspnoea and cough. The clinical problem is not so difficult for the GP, however, as chest X-rays are easily obtained, though there is the small risk of a negative X-ray even when lung cancer is present (Stapley *et al*, 2006b).

A similar message emerges for prostate cancer. The symptoms of cancer are largely the same as those of benign prostatic hyperplasia – with the exception of impotence. However, the ability to palpate the target organ by rectal examination makes diagnostics easier. Furthermore, a prostate-specific antigen (PSA) test is very good at identifying cancer in a man with lower urinary tract symptoms – indeed once the PSA result was added to the symptom models, it was the only feature still associated with cancer. This fits clinically: in a man with lower urinary tract symptoms, it is the PSA and the rectal examination that predict cancer, not the particular symptoms.

Values similar to the CAPER score could be calculated for lung and prostate cancer. The need for them is not so pressing, when both cancers have a primary care test with reasonable performance characteristics (the chest X-ray and PSA test, respectively). Any scoring system for lung cancer will have to be simpler than filling in an X-ray request form! It is very likely that the ovarian cancer results will lead to a scoring system, as the diagnosis appears so difficult, and the optimum investigation – transvaginal ultrasound – is not widely available.

In the brain tumour study, the main finding was the tiny 0.1% risk of a brain tumour with new-onset headache. This matched clinical experience, though, unfortunately, symptom recording in the General Practice Research Database (GPRD) was too infrequent to allow identification of second symptoms accompanying headache that added up to a high-risk combination.

Much of the above accords with current clinical practice. It was encouraging to see emerging from the results of these studies symptoms that were already known, even if the strength of the association with cancer was previously largely unknown. However, it is debatable if the clinical community is ready to move wholesale to a risk-based approach to selecting patients for rapid investigation. In the United Kingdom, entry to rapid investigation is guided by NICE guidance, which is (theoretically, at least) a risk-assessment schema. Should these new results simply be used to improve such guidance? This would certainly help, although there are many other aspects to primary care cancer diagnosis that are simply too subtle to be captured by research of this nature, let alone be expressed as a number. Furthermore, tricky decisions still need to be made about what level of risk warrants rapid investigation, and what doctors should do when they suspect cancer even though their patient does not fit guidance (Hamilton, 2009). Nonetheless, having a numerical value of risk of cancer can be helpful clinically, and is one step forward. Additionally, decision aids derived from work of this nature may be valuable, with a possibility of these being automated. It is technically feasible to programme practice computers to spot combinations of symptoms and alert the GP to a possible diagnosis, potentially allowing earlier diagnosis.

### The future

If we accept that risk estimates for primary care symptoms and cancer are valuable, then some logical conclusions can be drawn. There are 18 cancer sites with incidences over 50 new cases per million population each year. Of the four common ones, only breast is unstudied; of the medium incidence ones, ovary, uterus, lymphoma, melanoma and bladder, only ovary has been studied in any depth. The (difficult) choice is whether to use GP records directly as in the Exeter studies, and the recent ovarian study, or whether to use electronic databases. The latter are cheaper, have larger numbers and excellent recording of laboratory tests; they also are simpler in terms of ethical approval. Other teams have used them very successfully (Lawrenson *et al*, 2006; Jones *et al*, 2007). Size is important: it can allow subgroup analyses, such as the male and female anaemia results, which we have extended to calculation of age- and sex-dependent predictive values for the common colorectal cancer symptoms in the anaemia data set (Hamilton *et al*, 2009a). However, we found symptom recording to be better in paper records in a direct comparison (Hamilton *et al*, 2003). This is particularly important when examining multiple symptoms. A lesser advantage is the ability to code everything in the records, allowing unexpected features of cancer to be identified. However, the number of practices required (we used 39 in ovary) may make direct methods impossible logistically, so in all likelihood the rarer tumours will have to be studied by electronic methods.

## Figures and Tables

**Table 1:** Details of data sources for the CAPER studies

	**Colorectal**	**Lung**	**Prostate**	**Brain**	**Colorectal (anaemia)**
Data source	Direct from GP surgeries, including paper and computerised records			GPRD electronic	THIN electronic
Geography	All 19 Exeter practices			UK sample	UK sample
Year of diagnosis	1998–2002			1988–2006	2000–2006
Age range (years)	40 and over			18 and over	30 and over
Population size	60 500			Up to 4 000 000	Approx. 2 200 000
Number of cases	349	247	217	3505	3183

Abbreviations: GP=general practitioner; CAPER=Cancer Prediction in Exeter; GPRD=General Practice Research Database.

**Table 2:** Symptoms of cancer recorded in primary care, their prevalence in cases (%) and their likelihood ratios (LR)

**Colorectal cancer**			**Prostate cancer**			**Lung cancer**			**Brain tumours**		
**Symptom**	**%**	**LR**	**Symptom**	**%**	**LR**	**Symptom**	**%**	**LR**	**Symptom**	**%**	**LR**
Rectal bleeding	42	10	Urinary retention	15	9	Haemoptysis	20	13	New seizure	4	96
Loss of weight	27	5.1	Hesitancy	17	9	Loss of weight	27	6	Motor loss	9	21
Abdominal pain	42	4.5	Impotence	31	9	Loss of appetite	19	5	Confusion	3	16
Diarrhoea	38	3.9	Frequency	47	7	Dyspnoea	56	4	Weakness	3	11
Constipation	26	1.8	Nocturia	29	6	Chest or rib pain	42	3	Headache	10	7
			Haematuria	15	3	Fatigue	35	2	Memory loss	1	3
			Loss of weight	10	2	Cough	65	2	Visual disorder	1	3

*Note*: The first column shows the percentage of cases with the symptom recorded in their notes before diagnosis, and the second column is the positive likelihood ratio. For simplicity, all figures have been rounded to integers.

**Table 3:** Abnormal primary care investigations for cancer, their prevalence in cases (%) and their likelihood ratios (LR)

									**Anaemia in colorectal cancer**				
**Colorectal cancer**			**Prostate cancer**			**Lung cancer**				**Males**		**Females**	
**Result**	**%**	**LR**	**Result**	**%**	**LR**	**Result**	**%**	**LR**	**Haemoglobin (g dl^−1^)**	**%**	**LR**	**%**	**LR**
Positive FOB	9	31	PSA>4 ng ml^−1^	61	29	Thrombocytosis	14	19	<9.0	5	27	7	43
Haemoglobin 12–12.9 g dl^−1^	5	4	PSA>2 ng ml^−1^	61	19	Abnormal spirometry	10	14	9.0–9.9	4	17	5	12
Haemoglobin 10–11.9 g dl^−1^	11	4							10.0–10.9	4	7	8	7
Haemoglobin <10 g dl^−1^	12	10							11.0–11.9	5	4	8	3
Blood sugar>10 mmol l^−1^	7	3							12.0–12.9	6	3	10	2

Abbreviation: PSA=prostate-specific antigen.
